# Changes in organic carbon to clay ratios in different soils and land uses in England and Wales over time

**DOI:** 10.1038/s41598-022-09101-3

**Published:** 2022-03-25

**Authors:** Jonah M. Prout, Keith D. Shepherd, Steve P. McGrath, Guy J. D. Kirk, Kirsty L. Hassall, Stephan M. Haefele

**Affiliations:** 1 Department of Sustainable Agriculture Sciences, Rothamsted Research, West Common, Harpenden, AL5 2JQ Hertfordshire UK; 2 School of Water, Energy & Environment, Cranfield University, Cranfield, MK43 0AL Bedfordshire UK; 3 Innovative Solutions for Decision Agriculture (iSDA), Rothamsted Campus, West Common, Harpenden, AL5 2JQ, Hertfordshire UK; 4 Computational and Analytical Sciences Department, Rothamsted Research, West Common, Harpenden, AL5 2JQ Hertfordshire UK

**Keywords:** Environmental monitoring, Carbon cycle

## Abstract

Realistic targets for soil organic carbon (SOC) concentrations are needed, accounting for differences between soils and land uses. We assess the use of SOC/clay ratio for this purpose by comparing changes over time in (a) the National Soil Inventory of England and Wales, first sampled in 1978–1983 and resampled in 1994–2003, and (b) two long-term experiments under ley-arable rotations on contrasting soils in the East of England. The results showed that normalising for clay concentration provides a more meaningful separation between land uses than changes in SOC alone. Almost half of arable soils in the NSI had degraded SOC/clay ratios (< 1/13), compared with just 5% of permanent grass and woodland soils. Soils with initially large SOC/clay ratios (≥ 1/8) were prone to greater losses of SOC between the two NSI samplings than those with smaller ratios. The results suggest realistic long-term targets for SOC/clay in arable, ley grass, permanent grass and woodland soils are 1/13, 1/10, and > 1/8, respectively. Given the wide range of soils and land uses across England and Wales in the datasets used to test these targets, they should apply across similar temperate regions globally, and at national to sub-regional scales.

## Introduction

There is much interest in the potential for increasing the amounts of carbon held as organic matter in the world’s soils, both as a means of sequestering atmospheric CO_2_ and for improving soil properties generally. How realistic this is in practice is much debated, given the required large-scale changes in land management, the finite scope for SOC accumulation in any given soil, and the reversibility of increases in SOC stocks if land management or environmental conditions change^1–4^. Over millennial time scales, cultivation has caused losses of soil carbon of approx. 116 Gt C^5,6^, and soils are currently losing carbon in many parts of the world. For example, an analysis of data in the National Soil Inventory (NSI) of England and Wales found widespread losses of carbon from soils across both countries during the 1980s and 1990s^7^, mostly due to past changes in land management but possibly also linked to warming during that period^8,9^. On the other hand, soils in some regions show gains in carbon under both managed and natural vegetation^10–14^. Gauging realistic targets for SOC sequestration at local to national scales, and monitoring progress against targets, requires practicable indices which allow for governing factors and are based on readily measurable soil properties^3^.

Soil clay concentration is a key determinant of SOC concentration under given land use and environmental conditions. This is because SOC is protected from microbial attack by adsorption on clay-mineral surfaces and by occlusion within soil aggregates, and both of these are functions of clay concentration^15–17^. An analysis of 252 Polish arable soils, 305 French arable soils and 51 French pasture soils showed that a SOC/clay ratio of 1/10 marked the capacity for SOC protection by clay^18^. Soil physical conditions (bulk density, water retention, clay dispersibility) could be better explained by calculated properties of ‘complexed’ and ‘non-complexed’ SOC and clay. The 1/10 ratio corresponded to an approximate maximum for correlations between these and the soil physical properties. This idea was developed further and found, from an analysis of 161 Swiss arable and pasture soils, that SOC/clay ratios of 1/8, 1/10 and 1/13 marked boundaries between *Very Good*, *Good*, *Moderate* and *Degraded* levels of soil structure, respectively^19^. We have confirmed the value of an index of SOC status based on these threshold values for 3809 soils in the NSI of England and Wales under arable crops, ley grass, permanent grass and woodland^20^. Though highly weathered tropical soils with clay fractions dominated by sesquioxides may behave differently^21^, the index appears to work very successfully for a wide range of temperate soils across Northern Europe. By extension it should work in other temperate parts of the world.

In this paper we consider the use of the index for gauging changes in SOC over time under different land uses, and its use for assessing SOC status across different soil types under given land uses. We assessed changes in the SOC/clay index between the two samplings of the NSI of England and Wales over 12–25 years, during which both large losses and gains of SOC were found, largely due to historic changes in land management^8^. This is one of the most comprehensive datasets globally for this purpose, covering a wide range of soils and land uses, and with the same sampling and analytical methods used at both samplings. We also analyse changes in the index in more frequent sampling of two long-term experiments in contrasting soils under ley-arable cropping with different organic matter managements. Due to past cropping history, one of these experiments started at low SOC on a soil low in clay and the other started with a higher SOC and clay. We show how management systems that increase or decrease SOC can be normalised by using the SOC/clay index, and how long management changes take to bring about changes.

## Methods

### National soil inventory

The NSI was first sampled between 1978 and 1983. Topsoil (0–15 cm depth) samples were collected as 25 samples on a 20 × 20 m grid at each site. These sites were located at intersections of an orthogonal 5 km grid over the entire area of England and Wales, excluding urban areas and water bodies (www.landis.org.uk)^22^. Sufficient subsets of the sites (approximately 40% of the original sites) were resampled at intervals from 12 to 25 years after the original sampling to be able to detect changes in SOC concentration ≥ 2 g kg^−1^ with 95% confidence, taking account of the accuracy of the laboratory methods (± 1 g kg^−1^), the size of the original sampling and the distribution of SOC in the original sampling ^7^. This was done in three phases: in 1994–1995 for arable and ley grass sites (853 of the original 2578 sites), in 1995–1996 for managed permanent grassland sites (771 of the original 1579), and in 2003 for non-agricultural sites (including deciduous and coniferous woodland; 555 of the original 1505). Soil from both samplings was analysed using the standard methods of the Soil Survey of England Wales^23^: organic carbon by the Walkley–Black method, clay by the pipette method and pH in water at 1:2.5 soil:solution ratio. To check for differences in analytical precision between the samplings, stored samples from 10% of the original sites were reanalysed for SOC, and good agreement (± 1 g kg^−1^) with the original values was found across the full range of values^7^. How accurately the sites could be relocated was assessed by revisiting 10 sites following the original site descriptions and recording positions with a global positioning system; accuracy was better than 20 m on enclosed land and better than 50 m on open land^7^.

**Figure 1:**
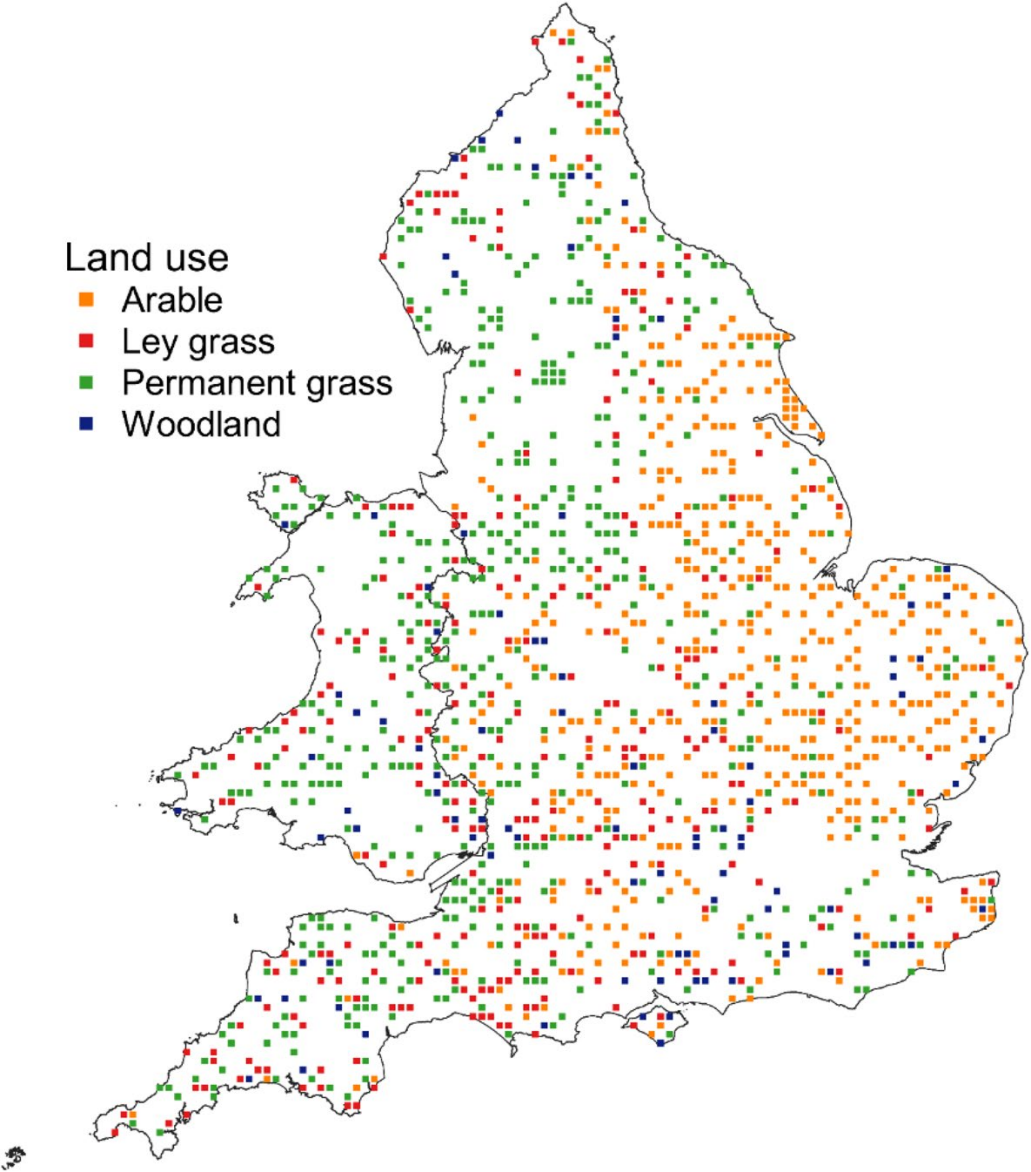
Distribution of National Soil Inventory sites selected for analyses (*n* = 1418). The map was produced using QGIS 3.0.1-Girona^25^.

For the analyses presented here, we only considered arable, ley grass, permanent grass and woodland sites which had the same land use at the two sampling dates. We excluded sites (a) classified as peat or organic as defined by SOC concentration > 120–180 g kg^−1^ for clay concentration 0–600 g kg^−1^^24^, (b) without measurements of clay concentration or pH, and (c) with SOC/clay ratio > 0.361 to agree with Prout et al.^20^. To allow for differences in sampling interval between sites, we adjusted SOC values in the second sampling for a notional sampling interval of 15 years, assuming the rate of change over the actual interval was constant. We calculated annual rates of change in SOC and SOC/clay ratio from the change in SOC between the samplings divided by the accurate time interval. Since clay concentrations were only measured on soils from the original sampling, we assumed no change between samplings. We excluded sites for which changes in SOC/clay per year were greater than 0.02. This gave 1418 sites, whose distributions across England and Wales are shown in Fig. 1.

We divided sites according to SOC/clay thresholds of 1/8, 1/10 and 1/13 as the boundaries between *Very Good*, *Good*, *Moderate* and *Degraded* levels of soil structure, respectively, following Johannes et al.^19^. In our earlier paper^20^ we showed that these thresholds were supported by the SOC/clay ratios of soils from the first NSI sampling that differed in structural condition, which we determined using a method derived from the Agricultural Land Classification of England and Wales^26^.

### Long-term experiments

Detailed land management practices were not recorded in the NSI, but the land use was the same at the two dates 12–25 years apart. To give more information on how more detailed changes in land management affect the SOC/clay index at more frequent time intervals, we used data from two long-term experiments under arable, ley grass and permanent grass. The two experiments have run over similar time periods, overlapping the period of the NSI samplings. One is on a sandy loam soil (Woburn) and the other on a silty clay loam (Harpenden).

#### Woburn ley-arable experiment

The Woburn experiment was established at Woburn Experimental Station, Woburn, Bedfordshire in 1938–1942. Details and treatments are in Table 1. We considered six treatments: arable (with or without fallows), lucerne (or sainfoin) converted to 3-year grass-clover ley, grazed ley converted to 3-year grass ley with inorganic nitrogen (N) additions, and alternating cycles of arable, lucerne, and grazed ley converted to 8-year leys (either grass-clover or with inorganic N). Farmyard manure (FYM) additions (fresh weight of 38 Mg ha^−1^ year^−1^) were applied to the first test-crop on one of the two paired-rotation plots in each experimental block until the mid-1960s; the SOC measurements of with- and without FYM plots were averaged for each treatment. Soils were sampled every fifth year. The SOC values used here are averages of five experimental blocks for each treatment. Only the first of the two 8-year ley treatment cycles was used for this analysis.

**Table 1:** Summary of treatments in the long-term experiments.

	Woburn		Highfield	
Location	52° 1′ 12″ N, 0° 37′ 12″ W		51° 80′ N, 00° 36′ W	
Establishment	1938–1942		1948 (1959 for bare fallow)	
Previous history	> 62 years of arable		> 100 years of permanent grass	
Soil^a^	Sandy loam, 114–164 g clay kg^−1^		Flinty silty clay loam, 233–335 g clay kg^−1^	
Structure	Three years of treatment crops followed by 2 years of test crops		Three years of treatment crops followed by 3 years of test crops	
Treatments	Arable (no fallows)	Arable crop rotations with 1-year hay in rotation until 1975	Arable	Arable crop rotations
	Arable (with fallows)	Arable crop rotations with 1-year root crop until 1975 and 1-year fallow until 1995	Bare fallow	Routinely ploughed and kept free of vegetation
	Lucerne/LC3^b^	Lucerne or sainfoin until 1975 after which 3-year grass-clover ley (LC3)	Grazed ley/LC3^c^	Changed from grazed ley to grass-clover ley (no inorganic nitrogen) from 1962
	Grazed ley/LN3^b^	Grazed ley until 1975 after which 3-year grass with inorganic nitrogen (LN3)	Cut grass/LN3^c^	Changed from cut-grass ley to grass-ley with inorganic N additions from 1962
	Alternating/LC8^b^ Alternating/LN8^b^	Alternating cycles (arable, lucerne, or grazed ley) until 1975 after which a 10-year structure of either 8-year grass-clover ley (LC8) or 8-year grass with inorganic N (LN8)	Old grass	Old grass was undisturbed pasture and reseeded grass was broken up and re-sown to long-term grass; 3-year cycles (2 years sheep grazing, 1 years hay with aftermath grazing); grazing was discontinued in 1962 (old grass) and 1963 (reseeded grass)

^a^Clay concentrations for Woburn from Catt et al*.*^27^, and for Highfield from Jensen et al*.*^28^.

^b,c^Change in treatment occurred at approximately the same time between samplings in all treatments of each experiment respectively.

#### Highfield ley-arable experiment

The Highfield experiment was established at Rothamsted Research (formerly Rothamsted Experimental Station), Harpenden, Hertfordshire in 1948. Details and treatments are in Table 1. We considered the following treatments: arable, ley grass, reseeded grass, old grass, and bare fallow. From 1961, FYM additions (fresh weight of 30 Mg ha^−1^ year^−1^) to sub-plots of potato crops were made to whole plots instead, then discontinued in 1970. The experiment was designed with four blocks and for each treatment the SOC/clay ratio was averaged across blocks. Incomplete sampling between years meant that the number of SOC measurements averaged was not always four. The bare fallow treatment comprised 4 plots, for which the SOC measurements per sampling year are averaged here. Where clay concentration was not measured directly in a plot, we used the clay concentration of the closest plot.

#### Changes in carbon stocks

Estimates of carbon stocks (Mg C ha^−1^ to 25 cm) were calculated for each SOC/clay threshold using the mean clay concentration of each long-term dataset (138 g kg^−1^ for Woburn, and 263 g kg^−1^ for Highfield), and the difference between each SOC/clay threshold (including a value of SOC/clay = 1/16, which emerged as a common ratio for long-term arable management at the two sites; see “Results”). For Woburn, a topsoil weight of 3770 t ha^−1^ was used for continuous arable soils^29^. The bulk density of Highfield soil was estimated to be 1.12 g cm^−3^ (topsoil weight = 2880 Mg ha^−1^) at the start of the experiment using starting SOC, texture measurements of Jensen et al.^28^, and a pedotransfer function for non-cultivated soils^30^ (the corresponding function for cultivated soils gave good agreement with the bulk density back-calculated from the soil weight for the arable soil of Woburn). We calculated equivalent soil masses so that changes in bulk density with SOC were accounted for. The standard deviations of SOC/clay values for each of the long-term experiments are presented in the Supplementary Tables S1, S2, and S3.

### Statistical analysis

R version 4.0.2^31^ was used to process the data and produce figures (package: ggplot2)^32^. Regression analysis was used to test how much of the variance in rate of change of SOC/clay could be explained by land use, average annual precipitation, major soil group, and mean pH between samplings. These variables were derived in the same way as Prout et al.^20^, except that average annual precipitation was averaged over 1910–2003 (extended to include the second sampling period). Empirical cumulative distribution functions, with pointwise bootstrapped 95% confidence intervals, were used to assess the difference in rate of change of SOC or SOC/clay by index class and land use. A chi-squared goodness of fit test was used to determine the representativeness of the smaller subset (n = 1418) compared to that of the larger subset in a previous study (n = 3809)^20^; the results are in Supplementary Table S4. Genstat^33^ was used to compare counts of index classes between the two time points of the subset (n = 1418) using a chi-squared test.

## Results

### National soil inventory

#### Distribution of SOC/clay

**Figure 2:**
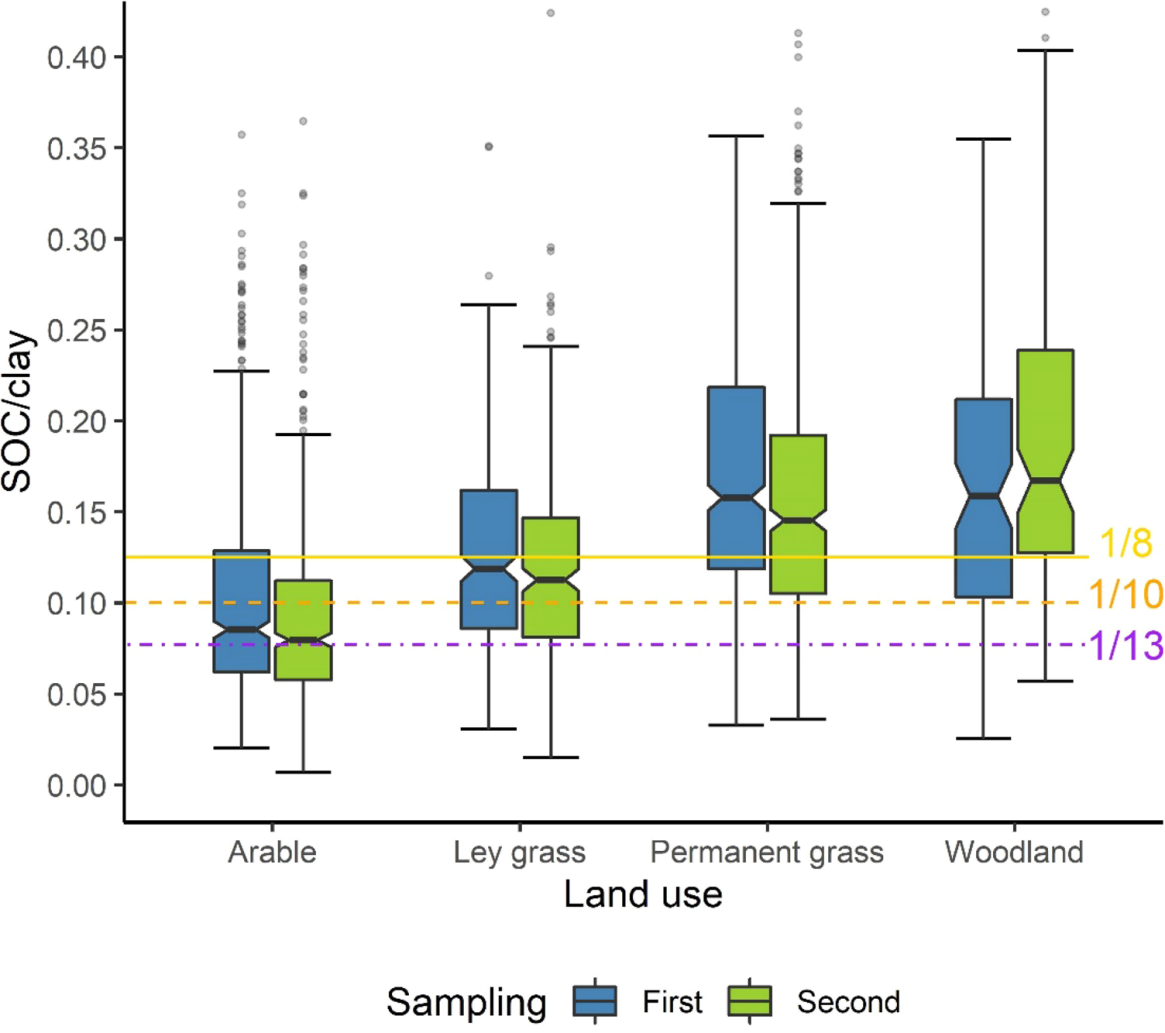
Boxplots of SOC/clay for each land use and sampling of the NSI. Notches extend to 1.58 × interquartile range/square-root(n) either side of the median and indicate 95% confidence around the median. Whiskers extend to the furthest point within 1.5 × interquartile range below the 25th-, or above the 75th percentiles. Observations outside of the whisker ranges are represented as points. The y-axis was truncated to 0.41 meaning that 11 points (across the data) are not visible.﻿

In both NSI samplings, SOC/clay ratios increased in the order arable < ley grass < permanent grass ≈ woodland (Fig. 2). However, the values tended to be smaller in the second sampling for arable, ley grass and permanent grass, and larger for woodland. The medians at each sampling date remained in the same index class for each land use respectively: *Moderate* (SOC/clay ≥ 1/13–1/10) for arable, *Good* (SOC/clay ≥ 1/10–1/8) for ley grass, and *Very Good* (SOC/clay > 1/8) for both permanent grass and woodland. However, the 25th percentile for each land use decreased to the next index class down for each land use (except for the second sampling of woodland which was also in the *Very Good* class). The specific changes in SOC/clay and index class are explored and analysed in the following sections.

#### Rates of change in SOC and SOC/clay

**Figure 3:**
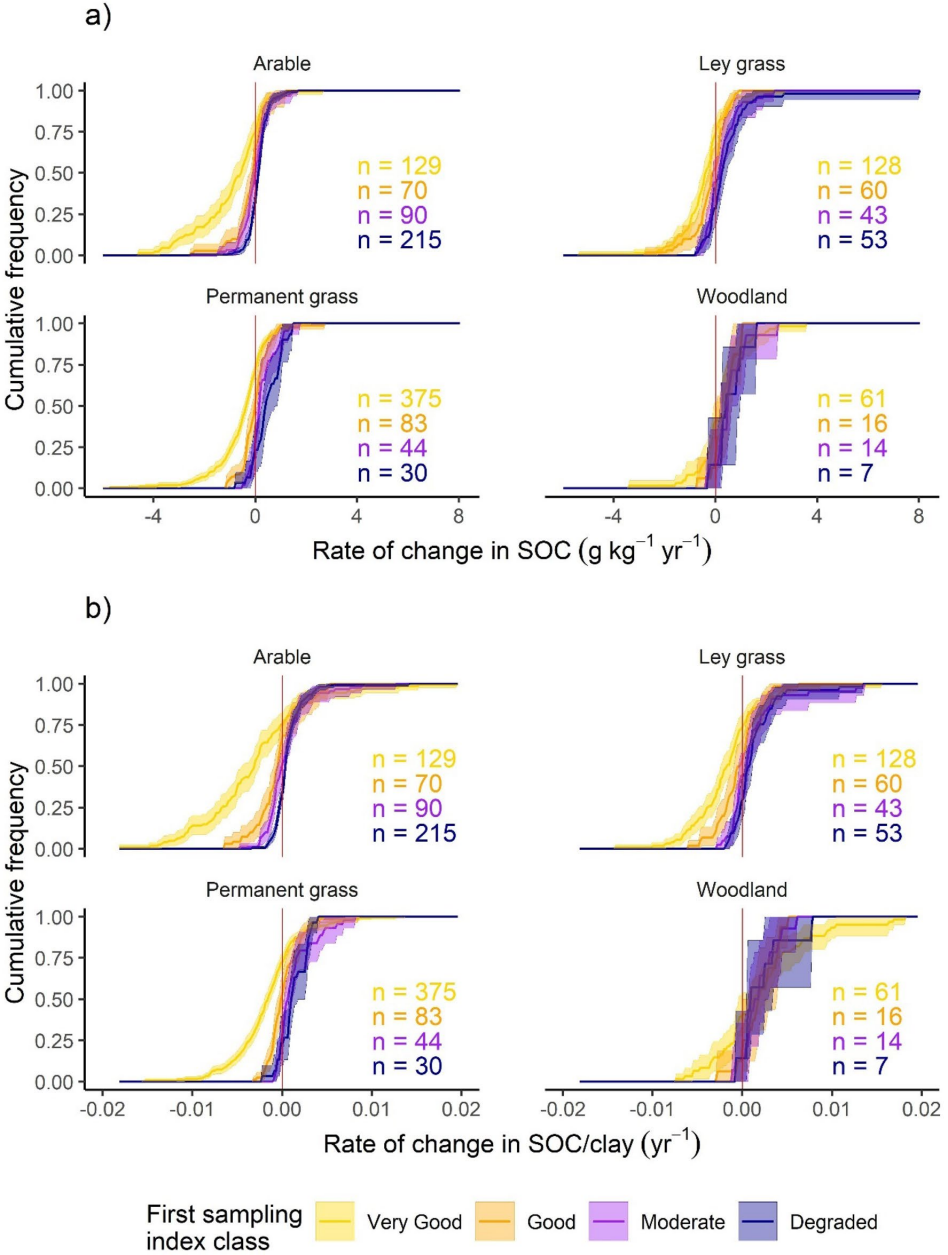
Empirical cumulative frequency distributions of change per year of (**a**) SOC, and (**b**) SOC/clay ratio in the two samplings of the National Soil Inventory, 1978–2003. Line colours of (**a**) and (**b**) indicate the SOC/clay index class from the first NSI sampling. Shaded areas represent bootstrapped 95% confidence intervals. Index classes correspond to SOC/clay ratios: *Very Good* ≥ 1/8, *Good* = 1/10–< 1/8, *Moderate* = 1/13–< 1/10, and *Degraded* < 1/13. Sample counts for the index classes of each land use are displayed in the corresponding colours. The vertical line indicates rate of change equal to zero.

The rates of change in SOC (Fig. 3a) and SOC/clay (Fig. 3b) were explored for each land use grouped by index class at the first sampling. The rates of change for both SOC and SOC/clay have the same cumulative frequencies of positive and negative rates for each index class (NB clay concentration did not change) and therefore the following applies to both. The *Very Good* class (SOC/clay > 1/8) had larger proportions of negative rates than the other index classes for all land uses except woodland where proportions were smaller. The other index classes had more similar curves to each other. In general, however, the proportion of negative rates followed the order of *Very Good* > *Good* > *Moderate* > *Degraded*. The proportions of the *Very Good* class with negative rates were similar for arable, ley grass and permanent grass (75, 73, and 73% respectively), however smaller proportions of negative rates were observed for all other index classes of ley and permanent grass compared with those of arable. Woodland soils mostly had positive rates of change, but negative rates were more frequent for *Very Good* soils.

The rates of change on the x-axis differ between Fig. 3a,b. The general trends between index classes for each land use were similar between the two, with the *Very Good* index class tending to have a greater range of negative rates than the other index classes. The difference between the two rates of change was most evident for the separation of the *Very Good* and *Good* curves, particularly those of ley grass. For the rate of change in SOC (Fig. 3a), the two means and respective confidence intervals overlapped, however for SOC/clay (Fig. 3b), the separation of the curves indicates a difference between classes. Considering the change in rate variable: the very high positive rate of change in SOC for the *Degraded* class of the ley grass data (8.0 g kg^−1^ year^−1^; Fig. 3a) was brought into line with the rest of the data when clay concentration was accounted for (Fig. 3b). The next highest rate of increase in SOC for *Degraded* ley grass soils was 2.7 g kg^−1^ year^−1^, in line with the highest rates for *Very Good* and *Moderate* ley grass soils.

Regression analysis was used to assess several factors other than land use, that might affect changes in SOC/clay. However, this showed that less than 1% of the variation in rate of change of SOC/clay could be explained by average annual precipitation, mean pH, major soil group or carbonate score. Because such a small amount of the variation was explained by these factors, this result was not considered further.

#### Changes in index class between samplings

The proportions of each land use falling in each index class are in Table 2. Arable had fewer soils classed as *Very Good* than *Degraded*, whereas permanent grass and woodland had a majority of *Very Good* soils, and ley grass was intermediate. The proportions in the subset of the NSI used in this analysis (*n* = 1418) were similar to those in the larger subset used in our earlier study^20^ (*n* = 3809) (Supplementary Table S4).

**Table 2:** Percentages of sites with a given index class under each land use in each sampling.

Land use	n	Percentage of sites with indicated SOC/clay index class							
		First sampling				Second sampling			
		Very Good	Good	Moderate	Degraded	Very Good	Good	Moderate	Degraded
Arable	504	25.6	13.9	17.9	42.7	20.7	12.5	20.0	46.8
Ley grass	284	45.1	21.1	15.1	18.7	41.2	17.6	21.1	20.1
Permanent grass	532	70.5	15.6	8.3	5.6	60.9	18.8	15.2	5.1
Woodland	98	62.2	16.3	14.3	7.1	77.6	12.2	5.1	5.1

The distribution of index classes by land use changed between the NSI samplings (*Χ*^2^(15) = 32.24, p < 0.001). From the first to the second sampling, the proportion of soils in the *Very Good* class decreased under arable and ley grass by close to 5%, and permanent grass by almost 10%. The increased proportion of the *Moderate* class under ley and permanent grass also contributed to the statistic. Arable soils in the *Degraded* class increased from 43 to 47%. In contrast, woodlands showed an increase in the proportion of the *Very Good* class and a decrease in the other index classes. The proportion of the *Very Good* class that changed index class followed the trend: arable > ley grass > permanent grass > woodland; the inverse was evident for the *Degraded* class (Table 3).

**Table 3:** Changes in index class between the two NSI samplings by land use and first sampling index class. Note the *Very Good* class could only move to a lower class and the *Degraded* class to a higher one, but the *Good* and *Moderate* classes could move either up or down.

Land use	Index class at first sampling			
	Very Good	Good	Moderate	Degraded
**Arable**				
Number at first sampling	129	70	90	215
Number that changed class	61	50	57	55
% that changed class	47	71	63	26
**Ley grass**				
Number at first sampling	128	60	43	53
Number that changed class	37	40	25	24
% that changed class	29	67	58	45
**Permanent grass**				
Number at first sampling	375	83	44	30
Number that changed class	86	51	25	18
% that changed class	23	61	57	60
**Woodland**				
Number at first sampling	61	16	14	7
Number that changed class	2	13	11	4
% that changed class	3	81	79	57

**Figure 4:**
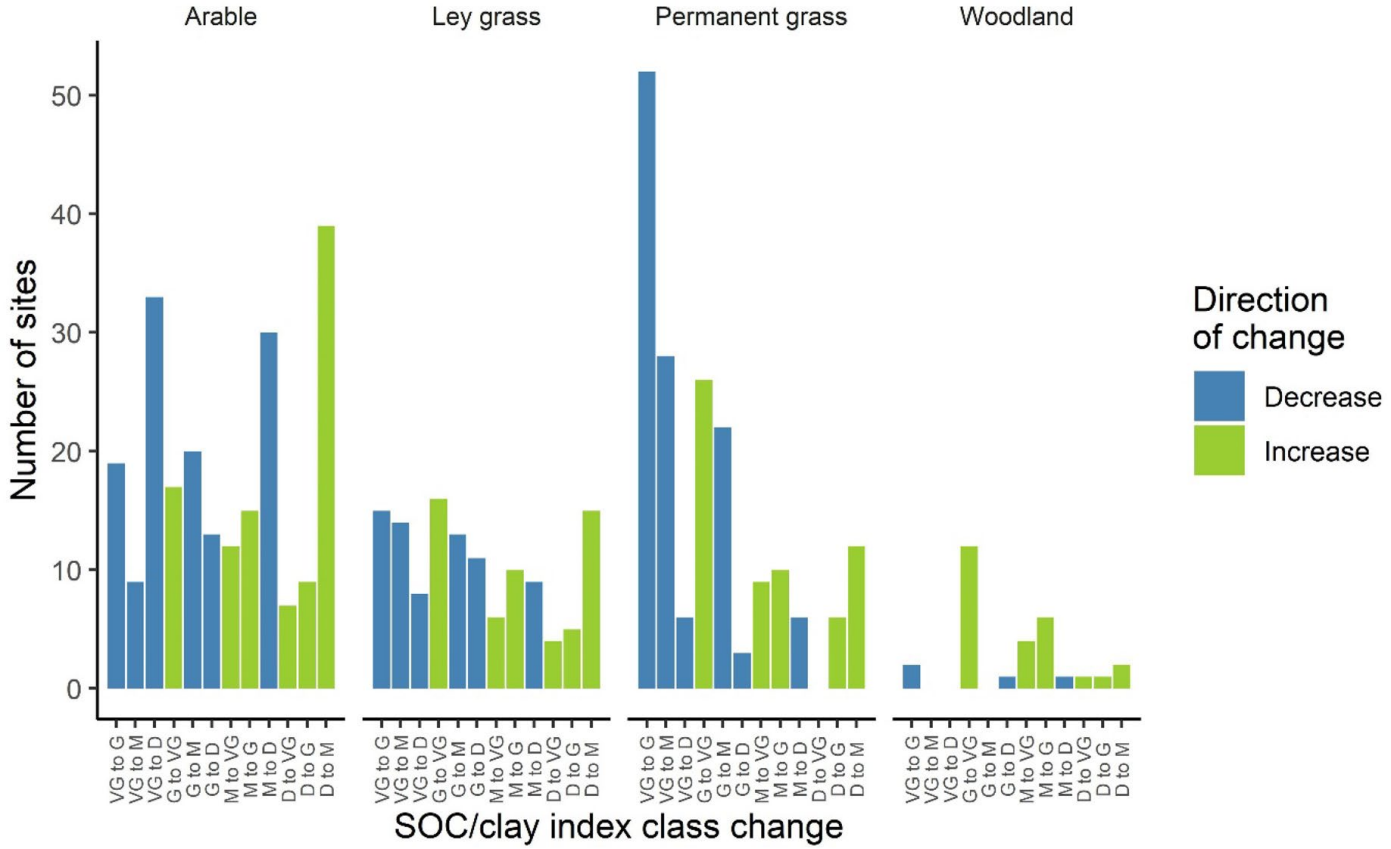
Numbers of sites that changed SOC/clay index class between the NSI samplings. Numbers of sites in each land use: arable, 223; ley grass, 126; permanent grass, 180; woodland, 30. VG, G, M and D indicate *Very Good* (SOC/clay ≥ 1/8), *Good* (SOC/clay 1/10–< 1/8), *Moderate* (SOC/clay 1/13–< 1/10), and *Degraded* (SOC/clay < 1/13) classes.

Despite having similar numbers in the *Very Good* class, 18% fewer of the ley grass changed class compared to arable. The extent of decreases from *Very Good*, increases from *Degraded*, and changes in either direction for *Good* or *Moderate* classes could also be seen for each land use (Fig. 4). More arable soils became *Degraded* than grassland soils, irrespective of initial class. A higher number of *Very Good* class soils changed to a lower class under permanent grass, but the changes tended to be to *Good* or *Moderate* classes, and permanent grass had a higher proportion remain *Very Good* than arable or ley grass. A larger number from the *Good* class moved down index classes than moved up under arable or ley, though under permanent grass the split was even. A larger proportion of the *Moderate* class became *Degraded* under arable than grassland. The number of *Degraded* sites increasing to another index class showed a similar trend for each of these three land uses, with few *Degraded* soils achieving *Good* status, and very few becoming *Very Good*. Though arable soils had the largest number of sites improving from *Degraded* to *Moderate* it also had the largest number of *Degraded* sites and the largest proportion remaining *Degraded*. Fewer woodland soils changed class and most of the changes were to a better class than when first sampled. As the woodland sites tended to be *Very Good* already, had a smaller sample size, and changes in index class tended to be upward, the smaller numbers changing class was to be expected.

### Long-term experiments

**Figure 5:**
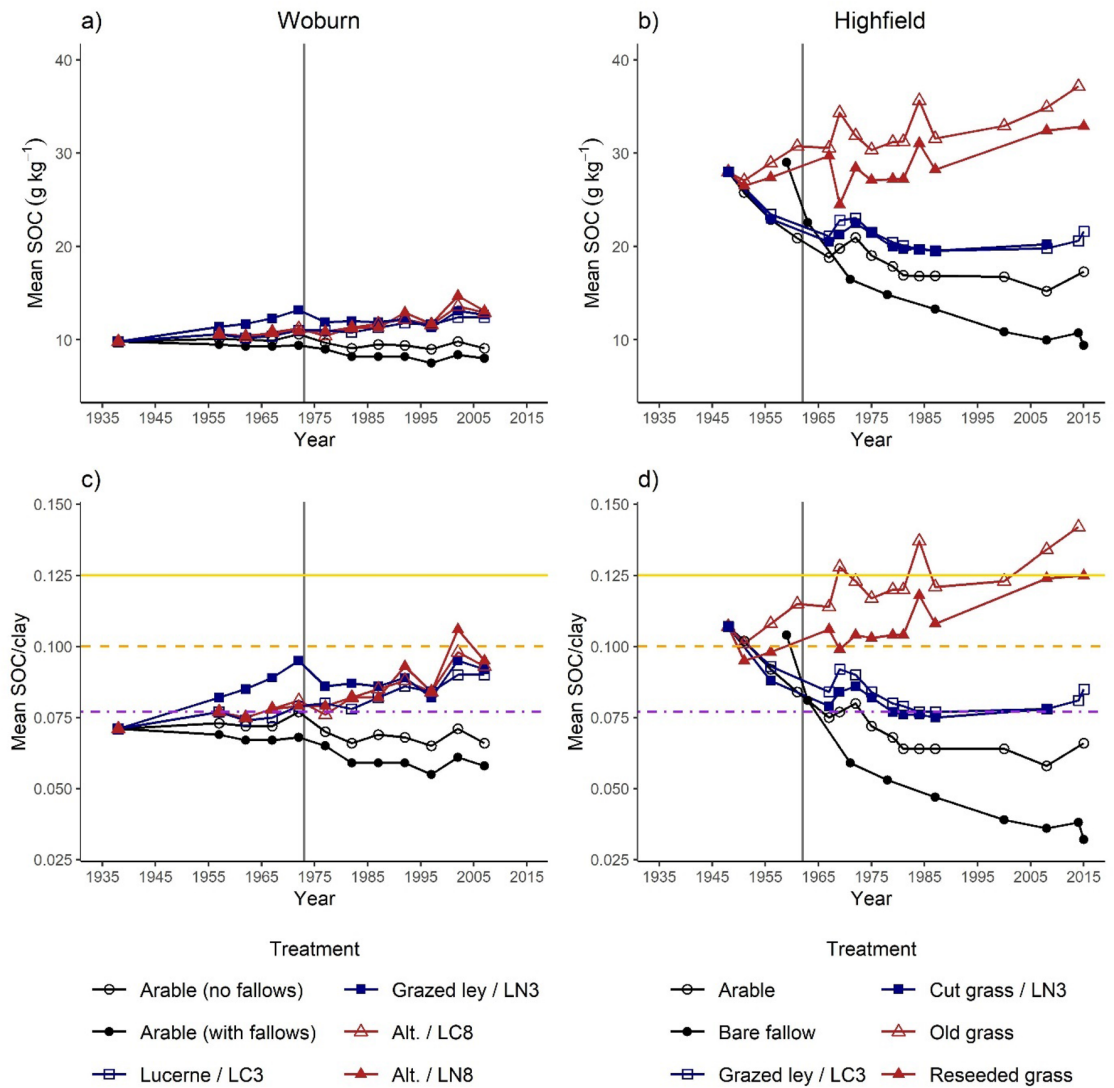
Mean SOC concentration and SOC/clay ratios over time in the long-term ley-arable rotation experiments at (**a**, **c**) Woburn (sandy loam) and (**b**, **d**) Highfield (silty clay loam). For standard deviations of each treatment at each timepoint see Supplementary Tables S1, S2, and S3. Horizontal lines represent SOC/clay index thresholds equal to 1/8 (solid), 1/10 (dashed), and 1/13 (dot-dash). The vertical line marks a change in treatments as indicated by the “/” in the legend entry. *Alt.* alternating treatment of 3-year arable, 3-year Lucerne, and 3-year grazed ley; *LC3* 3-year ley + clover; *LN3* 3-year ley + nitrogen; *LC8* 8-year ley + clover; *LN8* 8-year ley + nitrogen.

Figure 5a,b show the changes in SOC concentration over time in the two long term experiments on soils with contrasting clay contents. Woburn had been in long-term arable management and Highfield in long-term grass before the experiments began. Considering the flattening out of the curves for the ley and arable treatments from approximately 1980 onwards, SOC concentrations in the Woburn experiment were approximately half of those for similar treatments in the Highfield experiment. After normalising for clay concentration, similarities between the two sites are apparent. The SOC/clay results for each site are discussed below.

#### Woburn

Despite a history of arable management on the Woburn experiment site, SOC/clay ratio decreased over 70 years in the arable treatments, mostly remaining < 1/13, i.e. *Degraded*. The treatment without fallows started each treatment cycle with 1-year hay crop, which was evidently sufficient to maintain a higher SOC/clay ratio than the other arable treatment. After the hay rotation was stopped in 1975, SOC/clay decreased to approximately the level of the other arable treatment which decreased further from this point due to the introduction of fallow rotations. Apart from the 3-year grazed-ley (Grazed ley/LN3), which increased SOC/clay from *Degraded* to high in the *Moderate* class, the other treatments were similar to the arable treatment with 1-year hay for approximately the first 35 years (Fig. 5c). The two 8-year ley treatments were under an alternating rotation which included a grazed grass ley, but SOC/clay remained low, possibly due to the other treatments between grazed rotations. After the mid-1970s, however, the 3-year ley with clover (previously lucerne) and the 8-year leys showed similar, but more substantial increases. The peaks and troughs of the 8-year leys after 1970 correspond with samplings in the 8th and 3rd year under ley respectively. The replacement of grazing with inorganic N fertilizer resulted in a decline in SOC/clay for the grazed-ley treatment but it remained higher than the other ley treatments for approximately 15 year until they caught up. There was little difference between the shorter and longer leys from the 1990s onwards, though the shorter leys had lower SOC/clay in the 2000s, and little difference between the N-fertilized and clover leys of the longer rotations, both on a trajectory towards SOC/clay = 1/10 after the second phase of 8-year under ley grass. It is notable that in each case the 8-year leys decreased to a similar SOC/clay as the 3-year ley.

#### Highfield

Starting from long-term grass management and therefore a higher index class (SOC/clay > 1/10), SOC/clay ratios decreased under arable, ley grass, and bare fallow treatments, whereas they increased under grassland treatments (retained old grass or reseeded grass) (Fig. 5d). During the first 20 years, the index class in arable plots decreased from *Good* to the *Moderate*-*Degraded* threshold, then decreased further over the next 15 years before steadying at SOC/clay ≈ 1/16. This was similar to the Woburn arable treatments (mean SOC/clay across both Woburn treatments from 1982 to 2007 was 1/16), despite the Highfield soils having 110–160 g kg^−1^ higher clay concentrations. SOC/clay for the ley treatments decreased over the same periods as arable but remained in the *Moderate* class until around 1980. Between 1987 and 2008 there were no measurements in these treatments, but they still had SOC/clay close to the *Moderate*-*Degraded* threshold when next sampled, and the ley with clover showed further increases. The continuous bare fallow treatment started 10 years after the other treatments. However, within 4 years, SOC/clay decreased from *Good* to the level that arable and ley treatments took nearly 20 years to reach, and the soil was lower in the *Degraded* class than the arable soils within 10 years. The SOC/clay ratio continued to decline over the following 45 years. The bare fallow illustrates how far SOC can fall in this soil in the absence of crop inputs. The SOC concentration associated with the last samplings presented for bare fallow was approximately 10 g kg^−1^. This was comparable to, though slightly higher than, the arable treatments of Woburn which showed signs of equilibrating at 8–9 g kg^−1^. Probably due to the initial ploughing and reseeding, the SOC/clay ratio of reseeded grass decreased across the *Good*-*Moderate* threshold in the first 3 years followed by a general increase, approaching the *Very Good* class over 60 years. The SOC/clay of the old grass treatment showed an increasing trend over the course of the experiment, from the *Good* index class up to *Very Good* over 60 years. Both treatments were grazed until 1962–1963, which might explain some of the increase up to this point and subsequent plateau.

#### Carbon stocks

Using the clay concentrations of the long-term experiments as examples, the carbon stocks at each SOC/clay threshold and the difference that would result from a change in SOC/clay class (threshold to threshold) were calculated (Table 4). SOC/clay = 1/16 was included as a possible starting point for an arable scenario. Changes in SOC/clay equivalent to SOC/clay = 1/10 to 1/13 (or vice versa) were observed in both experiments. The ley treatments at Woburn gave rates of increase of 0.34–0.40 Mg C ha^−1^ year^−1^ (corresponding to 30–35 years). At Highfield, the loss of SOC from 1/10 to 1/13 in the bare fallow treatment was at a rate of 4.93 Mg C ha^−1^ year^−1^ compared with 0.985 Mg C ha^−1^ year^−1^ in the arable treatment. The increase in SOC/clay in the old grass treatment from 1/10 to 1/8 (from 1951 to 2008, 58 years) gave an overall rate of 0.317 Mg C ha^−1^ year^−1^.

**Table 4:** Carbon stock differences between SOC/clay thresholds for Woburn and Highfield soils. Carbon stock calculated to 25 cm depth.

Site	SOC/clay	Carbon stock at SOC/clay (t C ha^−1^)	Difference in carbon stocks between SOC/clay ratios (Mg C ha^−1^)		
			SOC/clay^a^		
			1/8	1/10	1/13
Woburn	1/8	65.0	–	–	–
	1/10	52.0	13.0	–	–
	1/13	40.0	25.0	12.0	–
	1/16^b^	32.5	32.5	19.5	7.5
Highfield	1/8	92.0	–	–	–
	1/10	73.6	18.4	–	–
	1/13	56.6	35.4	17.0	–
	1/16^b^	46.0	46.0	27.6	10.6

^a^Dashes indicate not applicable.

^b^Typical of arable management in the two experiments.

## Discussion

We have found that many of the arable, ley grass and permanent grass soils declined in SOC/clay between the NSI samplings, whereas many of the woodland soils improved. Except under woodland, many soils with initially *Very Good* and *Good* SOC/clay indices lost SOC between the samplings and moved to lower classes. Hence the proportions of *Moderate* and *Degraded* class soils increased, especially under arable and ley grass, and to a lesser extent under permanent grass. A previous study found that rates of SOC loss in the NSI at a given SOC stock decreased in the order arable > ley grass > permanent grass > other (mainly woodland)^8^. Those authors fitted a simple single-pool model with first order decomposition kinetics to the data. The model showed that, despite the diversity of soils in each land use, soils with small SOC stocks tended to gain C whereas those with larger values increasingly lost it, and there was a characteristic steady-state SOC stock for each land use under the prevailing management at which C was neither gained nor lost. The steady-state values increased in the order arable < ley grass ≈ permanent grass < other (mainly woodland), and the rate of gain or loss increased with the degree of departure from the steady-state SOC stock.

Since clay concentration is a major determinant of SOC protection and stability (Introduction﻿), the SOC/clay ratio gives a more definitive separation between different managements, climates and other factors within a land use category than SOC alone. Hence there was a clearer separation between the index values for cumulative plots of the rate of change in SOC/clay compared with SOC alone (Fig. 3). The SOC/clay ratio at which mechanisms of SOC stabilization are saturated is approximately 1/10^18^. Above this threshold soils are less likely to retain carbon under given OC inputs and below it they are more likely to retain added OC.

What were the causes of the declines in SOC/clay values between the NSI samplings and the differences between land uses? Past or continuing changes in land use and management were the major drivers and modelling results showed that the effects of the modest warming in the two countries between the samplings would not have been sufficient to explain much of the results ^8,9^. Likewise, atmospheric deposition of nitrogen or sulphur was not likely to have been a factor, at least in managed soils. There was roughly a 50% decrease in rainfall acidity between the NSI samplings as a result of decreased sulphur emissions from coal-fired power stations, leading to modest but widespread increases in soil pH across England and Wales^34^. However, there were no clear relationships between the SOC changes and either the baseline soil pH or measured changes in pH between the samplings^7^. Increased nitrogen deposition between the samplings may have increased SOC stocks on semi-natural land as a result of greater net primary production^14^. More recently, atmospheric nitrogen deposition in the UK has decreased^35^. Low uptake of reduced tillage practices over the sampling periods^36^ could also be a contributing factor to the declines in SOC/clay in arable soils.

Changes in land use and management will tend to shift SOC stocks towards new steady states, characteristic of these changes. This may cause gains of carbon in soils with small SOC stocks, or losses in soils with large stocks. There have been large changes in land use and management across England and Wales since the Second World War^37^. The following changes in particular will have affected SOC stocks: conversion of grassland and natural vegetation to crops after the war^38^; widespread improvements in land drainage^39^; greater use of mineral fertilizers^40^; greater animal stocking rates^41^; and, in general, the adoption of more uniform management practices across both countries^37^. Similar changes in land use and management affecting SOC stocks have taken place in other countries around the world and continue to do so^42,43^. Ambitions to increase SOC stocks globally need to be seen in the light of this.

The results of the long-term experiments illustrate the value of normalising for soil clay concentration. Sandy soils, such as those at Woburn, should not be expected to reach the same SOC concentrations as those with higher clay concentration, such as at Highfield. By quantifying SOC/clay ratios, the value of the SOC for soil properties can be assessed across contrasting soil types. Despite the differences in clay concentration between Woburn and Highfield (approximately 125 g kg^−1^), the arable treatments levelled off at a similar SOC/clay ratio of approximately 1/16. By contrast, the bare fallow treatment at Highfield had a similar SOC concentration to the arable treatment at Woburn (approximately 10 g kg^−1^) but had a much smaller SOC/clay ratio. Therefore, the bare fallow had a greater deficit of carbon and was degraded structurally and perhaps in other functions. This would not be apparent by comparing SOC values alone. Ley treatments had a moderating effect on SOC loss on conversion from grassland at Highfield and improved the soil from the *Degraded* class to near the *Good* threshold at Woburn. Results of the Woburn organic manuring experiment^20^—on the same site as the experiment presented here but with different treatments—largely agreed with these differences in index classes with SOC/clay for arable < 1/13 (~ 1/14), and ley > 1/13. Farmyard manure inputs gave greater increases in SOC/clay, but at unrealistically high rates. Combinations of these managements, such as through ley rotations with manure applications during arable rotations, might provide sustainable means to increase the rates and stability of SOC gains. Whilst the 8-year leys could achieve higher SOC/clay, they did not stabilise higher than the 3-year leys.

Key steps for soil management are soil testing to identify if a soil is *Degraded* (or better) followed by monitoring how management decisions impact the soil. Soil clay concentration is not expected to change, except with extreme abuse, so requires less frequent monitoring. Once clay concentration has been mapped, SOC can be monitored periodically to assess changes in the index. It is important that clay concentration is mapped accurately at the scale of interest, as shown by the large variability in the standard deviations of SOC/clay in the Woburn data (Supplementary Table S1). We expect that with more precise clay concentration data, as for Highfield, the mean SOC/clay results would be similar but with variability better accounted for.

Irrespective of how far soil carbon stocks might be increased globally to sequester atmospheric CO_2_, in many parts of the world soils needs to be restored to levels needed for resilience to the effects of climate change and continued provision of functions. Management practices for increasing SOC concentrations are well known and include management of manures and crop residues, use of cover crops and leys, and the various methods known as “Regenerative Agriculture”^44^. The long-term experiments reported here show that normalising for clay concentration allows more meaningful comparisons of management effects on SOC, and that our index SOC/clay ranges are of an appropriate magnitude for assessing changes over time. Very high SOC/clay ratios > 1/8 are unrealistic for all land uses, as shown in the various treatments in the long-term experiments and in the NSI results for the different land uses (Fig. 2). Arable soils had smaller SOC/clay ratios than permanent grassland and woodland soils.

Based on our results, we suggest suitable SOC/clay targets of > 1/13, > 1/10, and > 1/8 for arable, ley grass, and permanent grass and woodland soils, respectively. The net changes in SOC/clay between the NSI samplings for land uses other than woodland suggest that further decreases may have taken place, but the median SOC/clay in each sampling for each land use was still above these thresholds (Fig. 2). Timescales for achieving targets will depend on land use history, the availability of organic matter amendments, the length of ley rotations if employed, and practices such as the use of cover crops and reduced tillage.

In general, soils across England and Wales—and by extension managed soils in other temperate regions—have SOC concentrations below optimal levels due to sub-optimal management. The arable soils in the long-term experiments maintained a SOC/clay ratio ≈ 1/16, but could likely achieve SOC/clay ≥ 1/13 with improved management. On these soils, increasing SOC/clay to 1/13 could result in an increase of 6–9 Mg C ha^−1^ in the carbon stock. If this were to increase to SOC/clay = 1/10 that becomes 18–26 Mg C ha^−1^, which we have shown could be achievable with frequent or well managed ley rotations in approximately 40 years (Fig. 5).

A SOC/clay of 1/10 is proposed as an approximate limit for SOC protection by clay^18^, and we have shown that soils with SOC/clay > 1/8 are more likely to lose SOC, depending on land use. The results support the use of the SOC/clay ratio to normalise between different soils and inform management decisions to maintain SOC where possible, and increase it where needed.

## Conclusions

The SOC/clay ratio index provides a simple method to normalise SOC values across soils and to assess changes in SOC status over time. The NSI results showed that SOC/clay ratios declined between the NSI samplings under arable, ley grass and permanent grass, especially in soils that initially had large SOC/clay ratios. In arable land the proportion of *Degraded* soils increased between the samplings, even though a large proportion were already in that class. The results of the long-term experiments showed the value of normalising for soil clay when assessing SOC management practices. Similar management practices resulted in similar SOC/clay ratios on the two soils with contrasting clay concentration, despite SOC concentrations that differed by a factor of two. Realistic long-term targets for SOC/clay ratios differ between land uses: they are > 1/13, > 1/10, and > 1/8 for arable, ley grass, and permanent grass and woodland soils, respectively. Our conclusions are based on results for a wide range of soils across Northern Europe. While soils with different mineralogies may behave differently, for example, highly weathered soils of the humid tropics or volcanic ash soils, the basic SOC/clay index tested here should be useful in similar temperate regions globally.

## Supplementary Information

Supplementary Tables.

## Data Availability

The NSI dataset is held by Cranfield University and accessed via LandIS (www.landis.org.uk). Data for the Rothamsted ley-arable (Highfield) and Woburn ley-arable experiments can be obtained via the Electronic Rothamsted Archive (era.rothamsted.ac.uk).
