# Supplementary material for: Changes in organic carbon to clay ratios in different soils and land uses in England and Wales over time
Source: Sci Rep. 2022 Mar 25;12:5162. doi: 10.1038/s41598-022-09101-3 (PMC8956621; doi:10.1038/s41598-022-09101-3)
Supplement: Supplementary file 1 — Supplementary Tables. [file 41598_2022_9101_MOESM1_ESM.pdf]

Changes in organic carbon to clay ratios in different soils and land uses in England and Wales over time

By J. M. Prout et al

SUPPLEMENTARY INFORMATION

TABLE S1 Summary data of SOC/clay for the treatments of Woburn long-term experiment used to plot Figure 5 of the main paper. Mean and standard deviation (SD) represent five blocks each with two plots (one treated with farmyard manure for part of the experiment). The farmyard manure effect was not considered here.

	SOC/clay																	
	Arable (without fallows)			Arable (with fallows)			Lucerne / LC3			Grazed ley / LN3			Alternating / LC8[b]			Alternating / LN8[b]		
Year[a]	n	Mean	SD	n	Mean	SD	n	Mean	SD	n	Mean	SD	n	Mean	SD	n	Mean	SD
1938	10	0.071	0.006	10	0.071	0.006	10	0.071	0.006	10	0.071	0.006	10	0.071	0.006	10	0.071	0.006
1957	10	0.073	0.013	10	0.069	0.010	10	0.077	0.014	10	0.082	0.010	10	0.077	0.010	10	0.077	0.013
1962	10	0.072	0.011	10	0.067	0.011	10	0.074	0.013	10	0.085	0.012	10	0.075	0.009	10	0.075	0.012
1967	10	0.072	0.010	10	0.067	0.012	10	0.075	0.014	10	0.089	0.010	10	0.078	0.011	10	0.078	0.008
1972	10	0.077	0.011	10	0.068	0.009	10	0.079	0.014	10	0.095	0.011	10	0.081	0.012	10	0.079	0.009
1977	10	0.070	0.012	10	0.065	0.012	10	0.080	0.013	10	0.086	0.009	10	0.076	0.011	10	0.079	0.011
1982	10	0.066	0.012	10	0.059	0.012	10	0.078	0.009	10	0.087	0.013	10	0.082	0.009	10	0.082	0.011
1987	10	0.069	0.013	10	0.059	0.012	10	0.082	0.012	10	0.086	0.013	10	0.085	0.008	10	0.082	0.009
1992	10	0.068	0.011	10	0.059	0.012	10	0.086	0.013	10	0.089	0.014	10	0.088	0.006	10	0.093	0.014
1997	10	0.065	0.010	10	0.055	0.010	10	0.084	0.010	10	0.082	0.010	10	0.084	0.011	10	0.084	0.013
2002	10	0.071	0.013	10	0.061	0.012	10	0.090	0.013	10	0.095	0.013	10	0.098	0.010	10	0.106	0.016
2007	10	0.066	0.014	10	0.058	0.012	10	0.090	0.014	10	0.092	0.015	10	0.093	0.018	10	0.095	0.016

[a]After 1938, year is the midpoint of 5-year

[b]Values used are from the first cycle of this treatment. The second cycle started the second part of the treatment (LC8 or LN8) five years after the first cycle, but was not used in this analysis as it was not appropriate to merge the data and made the plot difficult to read if both were presented.

TABLE S2 Summary data of SOC/clay for the treatments of Highfield long-term experiment used to plot Figure 5 of the main paper. Mean and standard deviation (SD) represent four blocks each with a single plot (or two subplots, treated as one plot together here, for old grass and reseeded grass) per treatment.

	SOC/clay[a]														
	Arable			Old grass			Reseeded grass			Grazed ley / LC3			Cut grass / LN3		
Year	n	Mean	SD	n	Mean	SD	n	Mean	SD	n	Mean	SD	n	Mean	SD
1948	4	0.107	0.004	4	0.107	0.004	4	0.107	0.004	4	0.107	0.004	4	0.107	0.004
1951	2	0.102	0.003	2	0.101	0.011	2	0.095	0.002	-	-	-	-	-	-
1956	2	0.092	0.005	2	0.108	0.006	2	0.098	0.002	2	0.093	0.004	2	0.088	< 0.001
1961	2	0.084	0.001	2	0.115	0.009	-	-	-	-	-	-	-	-	-
1967	2	0.075	0.002	2	0.114	0.009	2	0.106	0.003	2	0.084	0.001	2	0.079	0.001
1969	1	0.077	-	1	0.128	-	1	0.099	-	1	0.092	-	1	0.084	-
1972	4	0.080	0.009	4	0.123	0.010	4	0.104	0.007	4	0.090	0.003	4	0.086	0.004
1975	4	0.072	0.008	4	0.117	0.011	4	0.103	0.008	4	0.084	0.005	4	0.082	0.006
1979	4	0.068	0.008	4	0.120	0.007	4	0.104	0.007	4	0.080	0.005	4	0.077	0.005
1981	4	0.064	0.007	4	0.120	0.009	4	0.104	0.007	4	0.079	0.005	4	0.076	0.005
1984	4	0.064	0.008	4	0.137	0.012	4	0.118	0.010	4	0.077	0.003	4	0.076	0.003
1987	4	0.064	0.006	4	0.121	0.010	4	0.108	0.009	4	0.077	0.005	4	0.075	0.006
2000	4	0.064	0.007	3	0.123	0.019	-	-	-	-	-	-	-	-	-
2008	4	0.058	0.005	4	0.134	0.013	4	0.124	0.006	4	0.078	0.004	4	0.078	0.005
2014	-	-	-	4	0.142	0.011	-	-	-	4	0.081	0.005	-	-	-
2015[b]	4	0.066	0.005	-	-	-	4	0.125	0.009	4	0.085	0.004	-	-	-

[a] Dashes (-) represent no data recorded (or not applicable in the standard deviation of year 1969 for which there was just one plot sampled).

[b] Each of the four plots (one per block per treatment) had three subsamples collected and measured in 2015. The mean and standard deviation were calculated at the plot-level (after taking the mean of the subsamples per plot).

TABLE S3 Summary data of SOC/clay for the bare fallow treatment of Highfield long-term experiment used to plot Figure 5 of the main paper. Mean and standard deviation represent four subplots within the bare fallow area.

	SOC/clay		
	Bare fallow		
Year	n	Mean	SD
1959	4	0.104	0.003
1963	4	0.081	0.002
1971	4	0.059	0.002
1978	4	0.053	0.003
1987	4	0.047	0.006
2000	4	0.039	0.004
2008	4	0.036	0.004
2014	4	0.038	0.004
2015[a]	1	0.032	-

[a]Mean was calculated from three samples of one subplot.

TABLE S4 Percentages of sites with a given index class under each land use in each subset of the first sampling of the NSI data.

		**Percentage of sites with indicated SOC/clay index class**								
		**Subset A (n = 3809)**				**Subset B (n = 1418)**				
**Land use**	**n**	**Very Good**	**Good**	**Moderate**	**Degraded**	**n**	**Very Good**	**Good**	**Moderate**	**Degraded**
Arable	1661	28.8	14.0	19.0	38.2	504	25.6	13.9	17.9	42.7
Ley grass	602	50.2	20.3	14.6	15.0	284	45.1	21.1	15.1	18.7
Permanent grass	1277	66.9	15.4	11.1	6.6	532	70.5	15.6	8.3	5.6
Woodland	269	67.7	16.0	10.8	5.6	98	62.2	16.3	14.3	7.1

Differences between the subsets of the first sampling of the National Soil Inventory in Prout et al. (2021), *Eur J Soil Sci*.; 72; 2493-2503. https://doi.org/10.1111/ejss.13012 (n = 3809) and this paper (n = 1418).
A chi-squared goodness of fit test was used to determine the representativeness of the smaller subset of the NSI (n=1418) compared to that of the larger subset in Prout *et al*. (2021) (n = 3809) ($X^2(9) = 17.61$, p = .04). The changes which contributed most to the difference were a decrease in the proportion of permanent grass soils in the *Moderate* class, an increase in the proportion of arable and ley grass soils in the *Degraded* class, and a lower proportion of arable and ley grass soils in the *Very Good* class. Despite these differences, the general trends were the same, with arable having fewer soil classed as *Very Good* than *Degraded* sites, contrasting with permanent grass and woodland, and ley grass having intermediate proportions (Table S1).
